# Comparison of Vitamin D Levels in Patients with and without Acne: A Case-Control Study Combined with a Randomized Controlled Trial

**DOI:** 10.1371/journal.pone.0161162

**Published:** 2016-08-25

**Authors:** Seul-Ki Lim, Jeong-Min Ha, Young-Ho Lee, Young Lee, Young-Joon Seo, Chang-Deok Kim, Jeung-Hoon Lee, Myung Im

**Affiliations:** 1 Department of Dermatology, School of Medicine, Chungnam National University, Daejeon, Korea; 2 Department of Anatomy, School of Medicine, Chungnam National University, Daejeon, Korea; University of Colorado Denver School of Medicine, UNITED STATES

## Abstract

**Background:**

Vitamin D plays an important role in the immune system, and its deficiency has been implicated in various skin diseases, including atopic dermatitis and psoriasis. Acne is a common inflammatory skin disease; however, the association with vitamin D remains unclear.

**Objectives:**

We evaluated vitamin D levels in patients with acne to determine the effect of vitamin D supplementation.

**Methods:**

This study included 80 patients with acne and 80 healthy controls. Serum 25-hydroxyvitamin D (25(OH)D) levels were measured, and demographic data were collected. Vitamin D-deficient patients were treated with oral cholecalciferol at 1000 IU/day for 2 months.

**Results:**

Deficiency in 25(OH)D was detected in 48.8% of patients with acne, but in only 22.5% of the healthy controls. The level of 25(OH)D was inversely associated with the severity of acne, and there was a significant negative correlation with inflammatory lesions. In a subsequent trial, improvement in inflammatory lesions was noted after supplementation with vitamin D in 39 acne patients with 25(OH)D deficiency.

**Limitations:**

Limitations of the study include the small number of patients in the supplementation study and the natural fluctuation of acne.

**Conclusions:**

Vitamin D deficiency was more frequent in patients with acne, and serum 25(OH)D levels were inversely correlated with acne severity, especially in patients with inflammatory lesions.

## Introduction

Acne is a common and complex skin disorder that distresses many patients because of its chronicity. Although multiple factors contribute to acne development, chronic inflammation is an important mechanism. Several inflammatory mediators such as cytokines, defensins, and neuropeptides have been identified in acne lesions.[1] In addition, *Propionibacterium acnes (P*. *acnes)* triggers cytokine activation by Toll-like receptors, which means that the innate immune system is also important for acne development.[2]

Vitamin D has a number of functions in addition to its well-known role as a modulator of calcium metabolism and homeostasis. It affects both the innate and adaptive immune system through its effects on T and B lymphocytes, dendritic cells, and macrophages,[3,4] and it is associated with systemic inflammatory diseases such as rheumatoid arthritis, systemic lupus erythematosus, and inflammatory bowel disease.[5,6] In dermatological diseases, it plays an important role as an immune modulator in atopic dermatitis, psoriasis, vitiligo, and alopecia.[7–10]

A few *in vitro* studies have published data that support the theory that vitamin D has a functional role in acne development. Identifying vitamin D receptors in human sebocytes and modulation of lipid and cytokine production by vitamin D suggest the possible association between vitamin D and acne pathophysiology.[11–13] However, evidence is lacking regarding the clinically relevant action of vitamin D in the development of acne, and its potential efficacy as a therapeutic agent in acne also requires clarification. Accordingly, we evaluated vitamin D levels in serum of acne patients compared to healthy controls, and the effects of vitamin D supplementation. To the best of our knowledge, this is the first study to have investigated the role of vitamin D in the pathogenesis and treatment of acne in a clinical setting.

## Materials and Methods

### Subjects

This case-control study included 80 patients with acne and 80 age- and sex-matched healthy controls. All of the patients and controls were enrolled in this study from November 2014 to February 2015 to avoid seasonal variation in vitamin D levels (recruitment end date, 28 February 2015; overall trial end date, 30 April 2015). Demographic data such as age, sex, body mass index (BMI), smoking history, and sunscreen use were collected prior to enrollment. Exclusion criteria prohibited enrollment of patients and controls who were receiving therapeutic interventions such as acne treatment, systemic corticosteroids, vitamin D supplements, or subjects with concomitant inflammatory diseases. This study was approved by the Institutional Review Board of Chungnam National University Hospital (CNUH 2014-07-013; date of approval, 24 July 2014). All of the subjects provided written informed consent before participating in the study. We did not include children in this study; written consent was obtained from the subjects themselves. This study was also approved by the ISRCTN registry (ISRCTN11007935; date of approval, 15 October 2015). Because we were initially unaware of the ISRCTN registry, the registration data were obtained after the study end date. The authors confirm that all ongoing and related trials for this drug/intervention are registered.

### Serum vitamin D analysis

Patients and controls had their serum 25-hydroxyvitamin D3 (25(OH)D) concentrations measured. Blood samples were collected from veins and analyzed within 24 h of sampling using the Roche Cobas e411 (Roche Diagnostics System, Switzerland). Levels of 25(OH)D were categorized as adequate (>20 ng/mL), inadequate (12–20 ng/mL), or deficient (<12 ng/mL) according to the guidelines set by the Food and Nutrition Board of the Institute of Medicine.[14]

### Vitamin D supplementation

**Fig 1:**
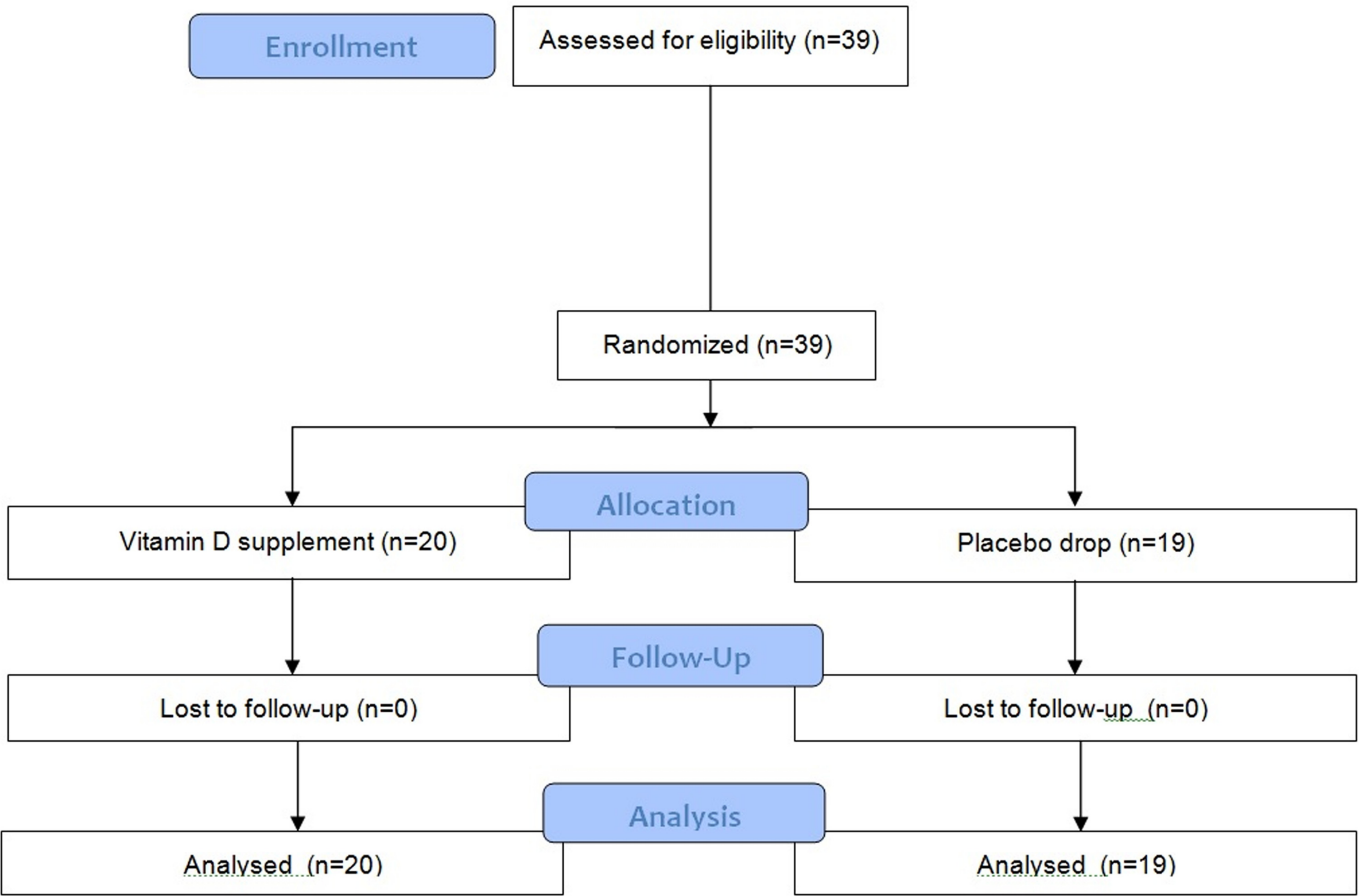
Flow diagram of randomized trial (CONSORT 2010 Flow Diagram).

A subsequent blinded controlled study was performed in acne patients with 25(OH)D deficiency. Each of the 39 patients were provided with a unique patient number and randomly assigned to one of two groups by computer: one group underwent 2-month oral administration of cholecalciferol (one drop of 1000 IU/day), and the other group received an identical-appearing placebo drop. Any other topical or systemic acne treatments, except for standard washing and moisturizing, were not allowed. The patients were assessed at the beginning of treatment and at 2, 4, and 8 weeks during treatment.(Fig 1)

### Clinical assessment

Clinical assessments were performed by three blinded independent dermatologists, and their objective assessments and inter-rater reliability were evaluated. Digital photographs at baseline and at each follow-up visit were used for the clinical assessments. Counts of non-inflammatory lesions (comedones) and inflammatory lesions (papules, pustules, and nodules) were also made at each visit.

The severity of acne was assessed according to the global acne grading system (GAGS) score.[15] GAGS divides the face, chest, and back into six areas (forehead, each cheek, nose, chin, chest, and back) and assigns a factor to each area on the basis of the surface area and distribution/density of pilosebaceous units. Each type of lesion is given a value depending on severity: no lesions = 0; comedones = 1; papules = 2; pustules = 3; and nodules = 4. The score for each area (local score) is calculated using the formula: Factor × Grade (0–4). The global score is the sum of the local scores, and acne severity is graded using the global score. A score of 1–18 is considered mild; 19–30, moderate; 31–38, severe; and >39, very severe.

### Statistical analysis

Statistical analyses were performed using SPSS version 15 (SSPS Inc., Chicago, IL). The chi-square test was performed to compare the categorical data (Tables 1 and 2). The correlation between the serum vitamin D level and inflammatory acne lesions was evaluated using Pearson’s correlation analysis. Changes in the vitamin D levels after vitamin D supplementation were evaluated with the Wilcoxon signed-rank test. The vitamin D levels according to disease severity and the median percentile changes from baseline in acne lesions after vitamin D supplementation were analyzed by the Kruskal–Wallis test followed by the Mann–Whitney U test for post hoc comparison. *P* values of <0.05 were considered statistically significant.

**Table 1:** Baseline demographic and clinical characteristics of patients with acne and controls.

	Acne (n = 80)	Controls (n = 80)	*P* value
**Age (years)**	20.9 ± 4.1	21.0 ± 5.7	0.768
**Sex (F/M)**, n (%)	47/33 (58.6/41.4)	41/39 (51.3/48.7)	0.482
**BMI (kg/m**^**2**^**)**	21.8 ± 3.8	20.2 ± 4.9	0.894
**Smoking (yes),** n (%)	16 (20.0)	11 (13.8)	0.645
**Using sunscreen**, n (%)	47 (58.8)	58 (72.5)	0.356
**Serum 25(OH)D (ng/mL)**	13.1 ± 9.8	15.2 ± 7.2	0.112
**Vitamin D deficiency**, n (%)	39 (48.8)	18 (22.5)	0.019*

All values are presented as mean **±** SD unless otherwise stated. *BMI*, body mass index; *25(OH)D*, 25-hydroxyvitamin D.

*P < 0.05

**Table 2:** Results of vitamin D deficiency according to influencing factors.

	Total	Mean 25(OH)D	Vitamin D Deficiency	*P* value
	no.	(ng/mL)	n (%)	
**Age (years)**				
<20	36	13.19 ± 2.8	20 (55.6)	
≥20	44	11.01 ± 1.8	19 (43.2)	0.624
**Sex**				
M	33	11.22 ± 3.8	17 (53.1)	
F	47	12.98 ± 3.2	22 (46.9)	0.892
**Duration (years)**				
<3	41	10.89 ± 1.8	24 (58.5)	
≥3	39	13.31 ± 2.2	15 (38.5)	0.211
**BMI (kg/m**^**2**^**)**				
Normal or less (<23)	50	10.89 ± 1.4	28 (56.0)	
Overweight or obese (≥23)	30	13.31 ± 1.2	11 (36.7)	0.189
**Family history**				
No	58	9.99 ± 1.4	30 (51.7)	
Yes	22	14.21 ± 0.5	9 (40.9)	0.503
**Smoking**				
No	64	10.44 ± 0.8	29 (45.3)	
Yes	16	13.76 ± 1.8	10 (62.5)	0.412
**Using sunscreen**				
No	33	13.51 ± 1.2	14 (42.4)	
Yes	47	10.69 ± 0.6	25 (53.2)	0.398
**Trunk lesions**				
No	54	15.56 ± 1.3	22 (40.7)	
Yes	26	8.69 ± 0.7	17 (65.3)	0.098
**Disease severity**				
Mild	27	17.37 ± 0.6	6 (22.2)	
Moderate	35	11.89 ± 1.0	18 (51.4)	
Severe	18	7.04 ± 0.5	15 (83.3)	0.002**

25(OH)D values are presented as mean **±** SD. *P* values are for comparison of vitamin D deficiency. *BMI*, body mass index; *25(OH)D*, 25-hydroxyvitamin D.

**P < 0.01

## Results

**Fig 2:**
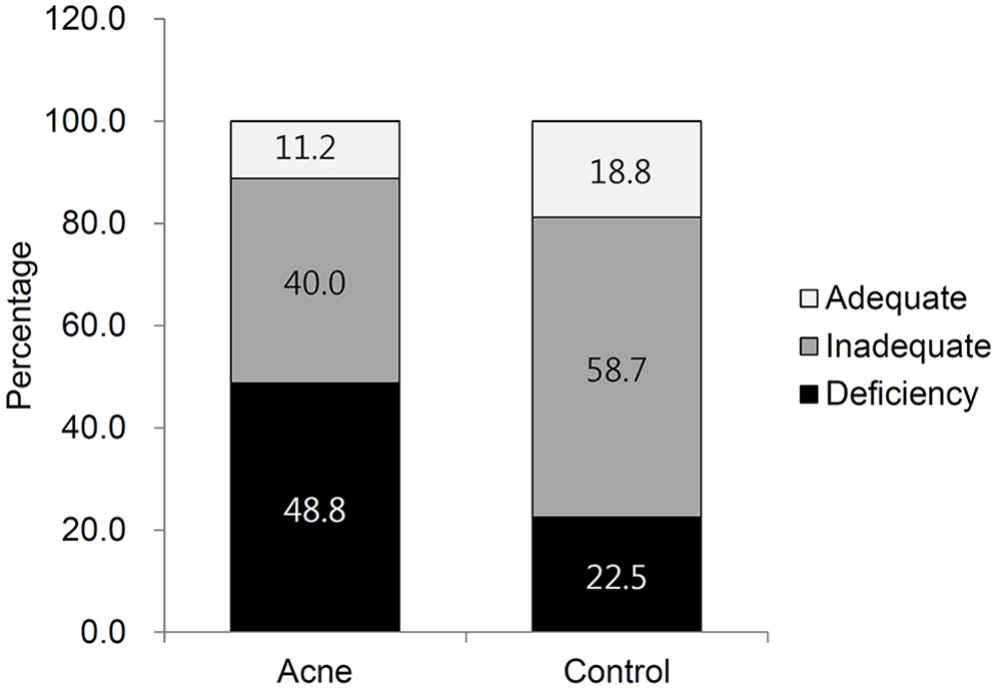
Percentages of patients with different vitamin D levels.

The age, sex, BMI, smoking history, and use of sunscreen were the same when comparing the two groups (Table 1). There were no significant differences in the mean 25(OH)D concentration between the groups, although it was lower in patients compared to the controls. However, the prevalence of 25(OH)D deficiency was significantly higher in patients with acne compared to healthy controls (48.8% vs. 22.5%; *P* = 0.019) (Table 1, Fig 2). The threshold of 25(OH)D deficiency in this study was defined as <12 ng/mL, according to the guidelines set by the Food and Nutrition Board of the Institute of Medicine.[14] The distribution pattern of the 25(OH)D levels in the two groups showed the widest gap in the 12-ng/mL area (S1 Fig).

**Fig 3:**
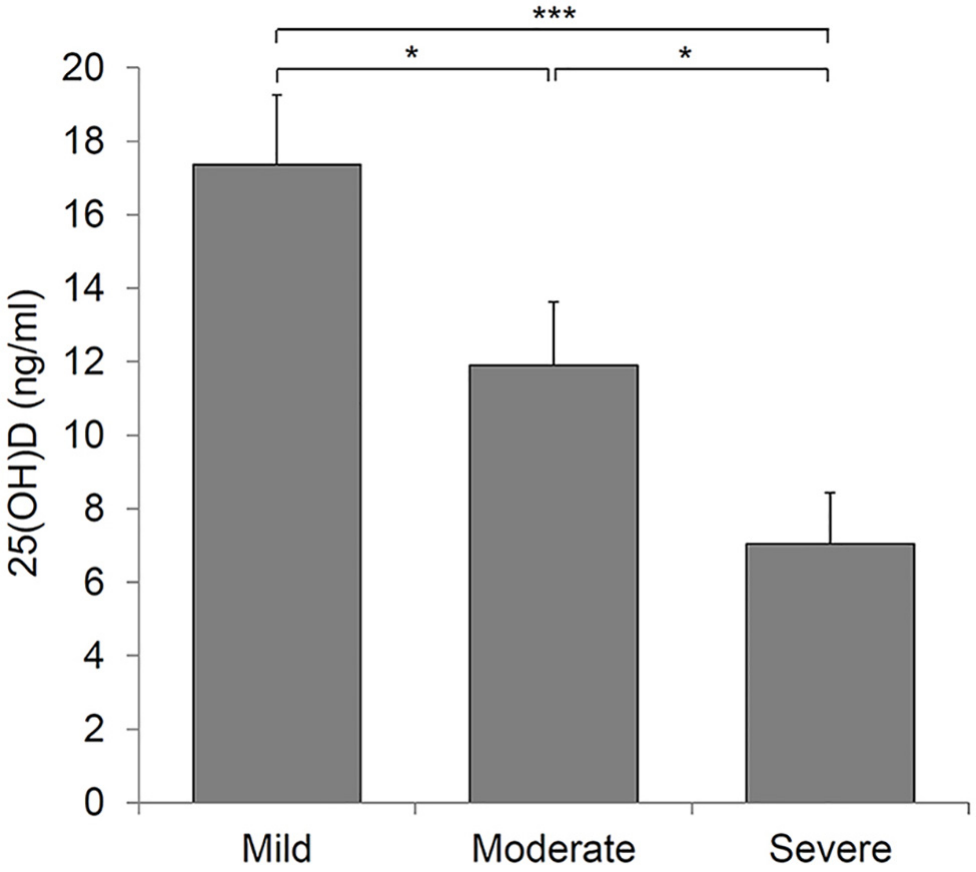
Vitamin D levels and disease severity (*P < 0.05, ***P < 0.001).

**Fig 4:**
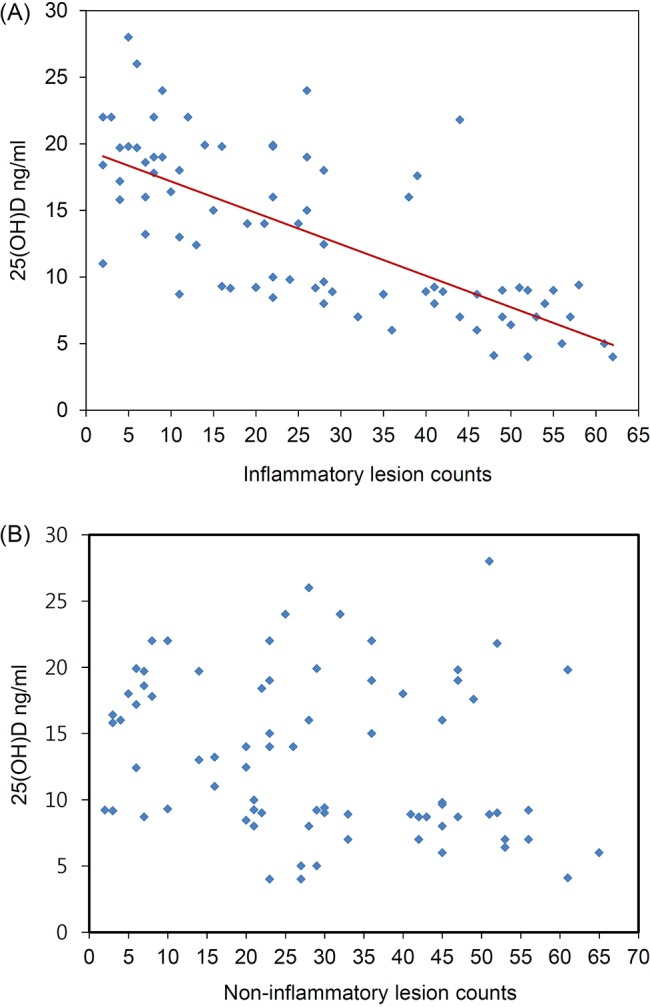
Correlation between vitamin D levels and inflammatory acne.

We determined whether vitamin D deficiency was influenced by any factor. No significant correlation was seen between deficient 25(OD)D levels and age, sex, disease duration, BMI, family history, smoking, sunscreen use, and trunk involvement. The only factor affecting 25(OH)D deficiency was disease severity. In total, 15 of the 18 patients (83.3%) in the severe group were 25(OH)D-deficient, whereas only 6 of the 27 (22.2%) patients in the mild group were deficient (Table 2). In addition, the mean 25(OH)D concentration was inversely associated with the severity of acne (Fig 3). There was not a significant correlation between 25(OH)D levels and the number of non-inflammatory lesions. However, the number of inflammatory lesions was significantly and negatively correlated with vitamin D concentrations (r = −0.512; *P* < 0.001) (Fig 4), signifying a possible link between the extent of vitamin D deficiency and the degree of acne inflammation.

**Fig 5:**
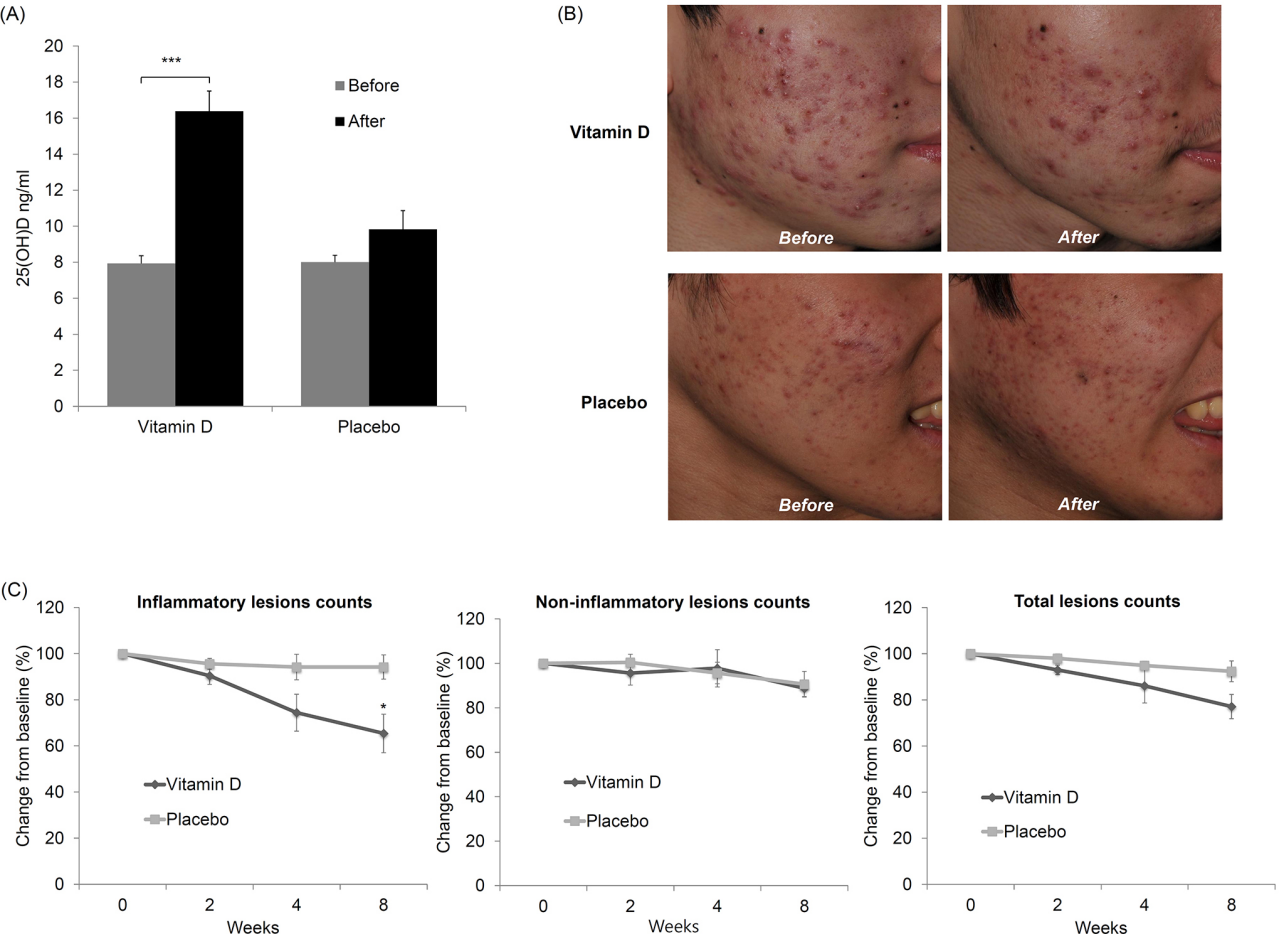
Clinical effects of vitamin D supplementation in patients with acne. (A) Change in vitamin D levels after vitamin D supplementation (***P < 0.001). (B) Photographs showing clinical improvement in a patient with acne. Photographs showing baseline (left) and last visit (right). (C) Median percentile changes from baseline in acne lesions (*P < 0.05).

We also assessed the therapeutic efficacy of vitamin D supplementation in acne patients with 25(OD)D deficiency. The 39 patients showing vitamin D deficiency were randomly assigned to the vitamin D (n = 20) or placebo (n = 19) group. The two groups did not significantly differ by any demographic or clinical factor. Vitamin D supplementation for 2 months resulted in a statistically significant increase in 25(OH)D levels (*P* < 0.001) (Fig 5A) and produced a clinical improvement compared to placebo (Fig 5B). There were no differences in the non-inflammatory and total lesion counts between the groups. However, the inflammatory lesions showed a statistically significant improvement in the vitamin D group compared with the control group (*P* < 0.05). Inflammatory lesions in the vitamin D group decreased by 34.6% after 8 weeks of treatment, whereas those in the control group decreased by 5.8% (Fig 5C). None of the patients reported discontinuation of the intervention, and there were no adverse events.

## Discussion

To the best of our knowledge, this is the first study to assess vitamin D status in acne patients. There were no significant differences in the mean vitamin D concentration between acne patients and healthy controls. This may be the result of the characteristics of vitamin D status in the Korean population. As shown in Fig 1, most healthy control subjects had inadequate levels of vitamin D, as reported previously in the general Korean population.[16–18] However, the prevalence of 25(OH)D deficiency was significantly higher in patients with acne (48.8%) compared to healthy controls (22.5%). A similar finding was reported in a previous clinical trial in which patients with nodulocystic acne showed relatively low serum vitamin D levels.[19]

To understand the vitamin D status associated with acne patients, we investigated the factors that influence vitamin D deficiency. Although obesity and decreased sun exposure using sunscreen are known to be associated with low 25(OH)D levels,[20–24] they were not associated with vitamin D deficiency in this study. The serum vitamin D level is also influenced by food such as fish oil or pork[16,17]; unfortunately, however, we were unable to evaluate the dietary habits of the patients. Our analysis revealed that the only factor associated with 25(OH)D deficiency was acne severity, similar to previous findings that disease severity of atopic dermatitis, psoriasis, and vitiligo is associated with lower levels of vitamin D.[7–9] Patients with severe acne may be subject to more psychological stress, and may tend to avoid spending extended periods outdoors, suggesting a possible explanation for low vitamin D levels in patients with severe acne.

In the randomized controlled trial of 39 acne patients with vitamin D deficiency, oral vitamin D supplementation produced a significant improvement in acne inflammation. In contrast, a previous study found no effect of vitamin D supplementation on acne lesions.[25] However, this result was due to the fact that patients with acne had polycystic ovary syndrome, and there was no consideration of the specific acne type, such as inflammatory lesions. The observed anti-inflammatory effects of vitamin D have several biological mechanisms. The expression of inflammatory biomarkers, such as interleukin (IL)-6, IL-8, and matrix metalloproteinase 9, is reduced by treatment with vitamin D in cultured sebocytes.[12] There is also published evidence that vitamin D inhibits *P*. *acnes*-induced Th17 differentiation with reduced expression of IL-17, an inflammatory cytokine that is increased in acne patients.[13] In addition, vitamin D has antimicrobial effects by inducing antimicrobial peptides such as LL-37 in human sebocytes.[26] These previous reports support the theory that vitamin D has an immune regulatory function in sebocytes, which supports the possible anti-inflammatory effects of vitamin D in acne patients.

Our vitamin D supplementation trial had a few potential limitations, such as the use of a low dose and short duration of treatment. The daily dose of vitamin D in this study was 1000 IU/day, lower than in previous studies.[27,28] However, some studies have shown that a daily dose of 1000 IU vitamin D is an effective treatment for atopic dermatitis.[29,30] In addition, as shown in Fig 4, the vitamin D level was significantly improved by 1000 IU/day for 2 months, although it was an inadequate level. Future trials need to examine the impact of regimens that are more likely to achieve adequate levels of vitamin D, which is often associated with optimal health. Moreover, given the frequent disease fluctuations that characterize acne, future trials of more patients with a longer treatment duration are needed to determine if acne lesions recur after the initial improvement or if the benefits are sustained by longer duration of treatment.

In conclusion, we found that vitamin D deficiency was more frequent in patients with acne, which was inversely correlated with disease severity, indicating a potential role of vitamin D deficiency in acne pathogenesis. A further study with a larger sample size is needed to confirm our results because of the small number of patients in the supplementation study and the natural fluctuation of acne. Evaluation of the tissue level of vitamin D in patients with acne will also require a further study to reveal direct evidence of the effect of vitamin D on acne.

## Supporting Information

S1 Fig Distribution pattern of 25(OH)D levels in patients and controls. The horizontal axis represents the 25(OH)D levels (ng/mL), and the vertical axis represents the cumulative frequency of 25(OH)D levels (%).
(JPG)

S1 File CONSORT 2010 Checklist. (DOC)

S2 File Study information and protocol. (DOCX)

S3 File Study information and protocol (Original language). (DOCX)
