# Supplementary figures and images for: Comparison of Vitamin D Levels in Patients with and without Acne: A Case-Control Study Combined with a Randomized Controlled Trial
Source: PLoS One. 2016 Aug 25;11(8):e0161162. doi: 10.1371/journal.pone.0161162 (PMC4999291; doi:10.1371/journal.pone.0161162)

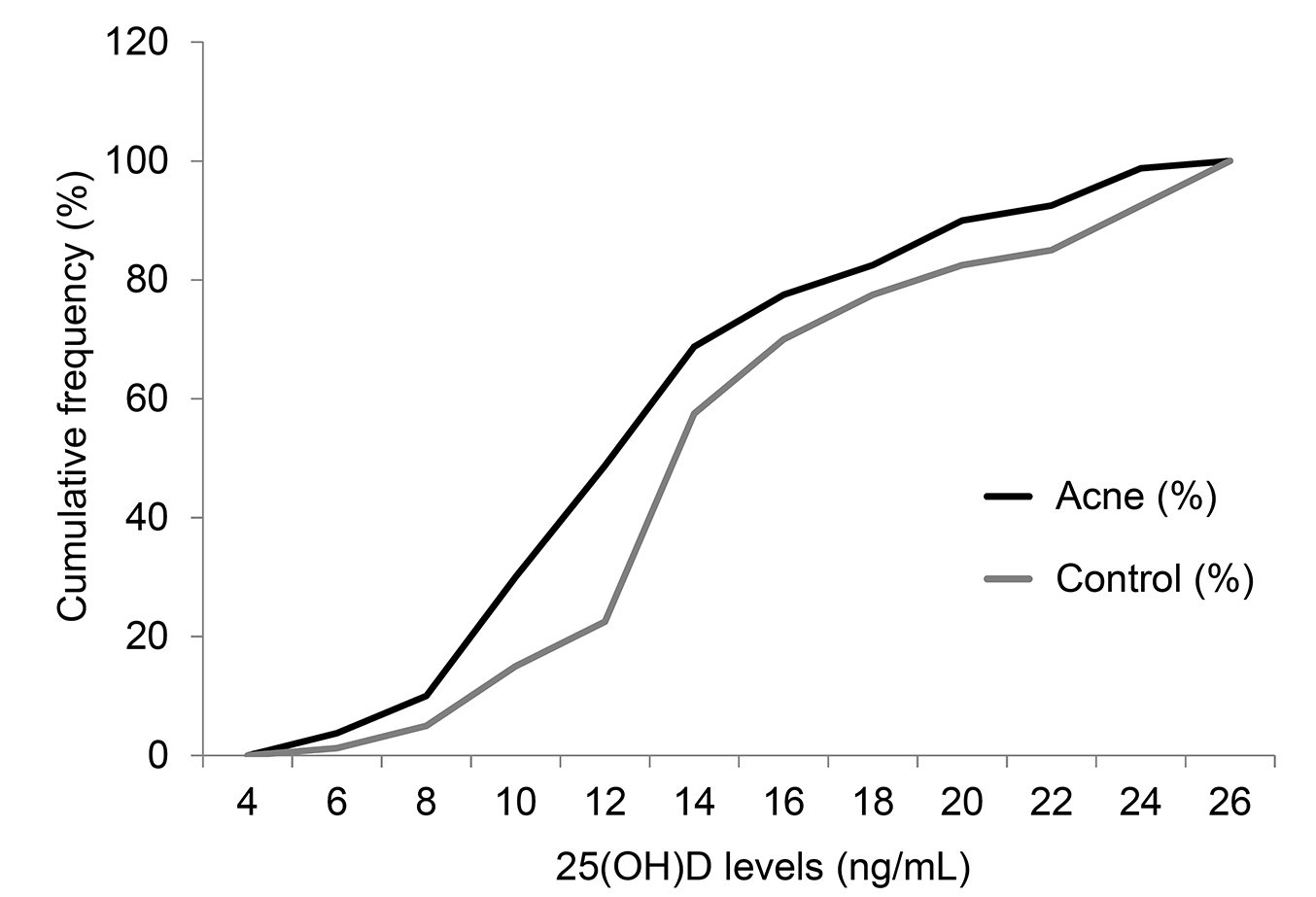

Supplement: S1 Fig — The horizontal axis represents the 25(OH)D levels (ng/mL), and the vertical axis represents the cumulative frequency of 25(OH)D levels (%). (JPG) [file pone.0161162.s001.jpg]
