# Supplementary material for: Comparison of Vitamin D Levels in Patients with and without Acne: A Case-Control Study Combined with a Randomized Controlled Trial
Source: PLoS One. 2016 Aug 25;11(8):e0161162. doi: 10.1371/journal.pone.0161162 (PMC4999291; doi:10.1371/journal.pone.0161162)
Supplement: S2 File — (DOCX) [file pone.0161162.s003.docx]

**Study information and protocol**

**Title**

Functional role of vitamin D in patients with acne

**Trial participating centre**

Chungnam University Hospital
282, Munhwa-ro Jung-gu
Daejeon
35015
Korea, South

**Chief of research**

Myung Im

Department of dermatology, Chungnam University Hospital

1. **The background of this study**

Acne is a common and complex skin disorder that distresses many patients because of its chronicity. Although multiple factors contribute to acne development, chronic inflammation is an important mechanism. Several inflammatory mediators such as cytokines, defensins, and neuropeptides have been identified in acne lesions. In addition, Propionibacterium acnes (P. acnces) trigger cytokine activation by Toll-like receptors, which means that the innate immune system is also important for acne development.

Vitamin D has a number of functions in addition to its well-known role as a modulator of calcium metabolism and homeostasis. It affects both the innate and adaptive immune system through its effects on T and B lymphocytes, dendritic cells, and macrophages, and it is associated with systemic inflammatory diseases such as rheumatoid arthritis, systemic lupus erythematosus, and inflammatory bowel disease. In dermatological diseases, it plays an important role as an immune modulator in atopic dermatitis, psoriasis, vitiligo, and alopecia.

1. **The purpose of this study**

Vitamin D plays an important role in the immune system and its deficiency has been implicated in various skin diseases, including atopic dermatitis and psoriasis. Acne is a common inflammatory skin disease; however, the association with vitamin D remains unclear.

To evaluate vitamin D levels in patients with acne to determine the effect of vitamin D supplementation.

1. **Trial participating centre**

**Trial participating centre**

Chungnam University Hospital
282, Munhwa-ro Jung-gu
Daejeon
35015
Korea, South

**Chief of research**

Myung Im

Department of dermatology, Chungnam University Hospital

**The person in charge**

Seulki Lim

Department of dermatology, Chungnam University Hospital

1. **Condition**

Acne (diagnosed clinically)

1. **Inclusion & Exclusion criteria**

**Target number of participants**

This study includes 80 patients with acne and 80 healthy controls.

**Participant inclusion criteria**

1. Adults aged between 20 and 35 years with acne
2. Adults aged between 20 and 35 years without acne (healthy controls)

**Gender**

Not Specified

**Participant exclusion criteria**

1. Receiving therapeutic interventions such as acne treatment, systemic corticosteroids, vitamin D supplements
2. Subjects with concomitant inflammatory diseases

1. **The study period**

**Overall trial start date**

01/11/2014

**Recruitment start date**

01/11/2014

**Recruitment end date**

28/02/2015

**Overall trial end date**

30/04/2015

**Overall data analysis end date**

31/05/2015

1. **The methods of study**

**Study design**

Intervention and Randomised controlled trial

**Primary study design**

Interventional

**Secondary study design**

Randomised controlled trial

**The protocol of this study**

**Data collection**

Demographic data such as age, gender, body mass index (BMI), smoking history, and sunscreen use will be collected prior to enrollment.

**Primary study design**

Acne patients (n=80) and healthy controls(n=80) have a blood test so that their serum 25-hydroxy vitamin D (25(OH)D) concentrations can be measured. Blood samples are collected from veins and analyzed within 24 h of sampling using the Roche Cobas e411 (Roche Diagnostics System, Switzerland).

**Secondary study design**
The acne patients who had vitamin D deficiency in blood test will be randomly assigned to either a 2 month oral administration of cholecalciferol (one drop of 1000 IU/day) or an identical-appearing placebo drop. Any other topical or systemic acne treatments, except for standard washing and moisturizing, are not allowed.

1. **Outcome meseures**

**Primary outcome measures**

Serum vitamin D level in patients with acne and healthy controls is measured using blood analysis of 25-hydroxy vitamin D (25(OH)D) concentration at baseline. Levels of 25(OH)D are categorized as adequate (> 20 ng/mL), inadequate (12–20 ng/mL), or deficient (< 12 ng/mL) according to the guidelines set by the Food and Nutrition Board of the Institute of Medicine.

**Secondary outcome measures**

1. The severity of acne will be assessed using digital photographs and the global acne grading system (GAGS) score at baseline, 2, 4 and 8 weeks. GAGS divides the face, chest, and back into six areas (forehead, each cheek, nose, chin, chest, and back) and assigns a factor to each area on the basis of the surface area and distribution/density of pilosebaceous units. Each type of lesion is given a value depending on severity: no lesions = 0; comedones = 1; papules = 2; pustules = 3; and nodules = 4. The score for each area (local score) is calculated using the formula: Factor × Grade (0–4). The global score is the sum of the local scores, and acne severity is graded using the global score. A score of 1–18 is considered mild; 19–30, moderate; 31–38, severe; and > 39, very severe.
2. Counts of non-inflammatory lesions (comedones) and inflammatory lesions (papules, pustules, and nodules) will be made at each visit, and dermatological assessments will be performed blind by three independent dermatologists.
3. **DATA analysis**

Statistical analyses will be performed using SPSS version 15 (SSPS Inc., Chicago, IL). The Mann–Whitney U-test will be used for post hoc analysis. To compare the categorical data, the Chi-square test or the Fisher’s exact test will be performed. Correlations will be performed using the Spearman’s correlation analysis. P values < 0.05 are considered statistically significant.

1. **Publish**

After finishing data analysis, we will submit the article to journal of dermatology.

1. **References**

- Vitamin D status in patients with rosacea. O¨zlem Ekiz et al. Cutan Ocul Toxicol, 2014; 33(1): 60–62

- Serum 25-hydroxyvitamin D deficiency in chinese patients with vitiligo: a case-control study. Xu X et al. PLoS One 2012;7:e52778

- Vitamin D: emerging roles in infection and immunity. Bartly J. et al. Expert Rev Anti Infect Ther 2010;8:1359–1369

- A pilot study assessing the role of 25 hydroxy vitamin D levels in patients

with vitiligo vulgaris. Silverberg JI et al. J Am Acad Dermatol 2010;62:937–941
