# Supplementary material for: Comparison of Vitamin D Levels in Patients with and without Acne: A Case-Control Study Combined with a Randomized Controlled Trial
Source: PLoS One. 2016 Aug 25;11(8):e0161162. doi: 10.1371/journal.pone.0161162 (PMC4999291; doi:10.1371/journal.pone.0161162)
Supplement: S3 File — (DOCX) [file pone.0161162.s004.docx]

**연구 계획서**

연구제목:

(국문) 여드름 환자에서 비타민D의 기능

(영문) Functional role of vitamin D in patients with acne

연구기관 :**충남대학교병원**

연구기관장 :**원장 김봉옥**

연구책임자 : **임명**

소속: **충남대학교병원 피부과**

**1. 연구배경**

| 여드름(Acne vulgaris)은 흔하지만 복잡한 피부문제로 만성적인 질환이기 때문에 많은 환자들에게서 스트레스를 유발한다. 많은 요인들이 여드름 발생에 영향을 미치며, 그 중에서도 만성적인 염증반응은 여드름 발생에 중요한 기전이다. 다양한 종류의 염증 매게 물질(사이토카인, 디펜신, 뉴로팹타이드)이 여드름 병변에서 확인된다. 또한 프로피오니박테리움(Propionibacterium acnes)은 Toll-like receptor를 통하여 사이토카인을 활성화 시키면 이 과정에서 선천면역계가 영향을 미친다.  비타민 D는 칼슘 대사와 항상성 유지에 관여하는 것으로 알려진 물질로 이러한 작용 외에도 다양한 기능이 있다. 비타민 D는 선천, 후천 면역반응에 영향을 미쳐서 T세포, B세포, 수지상세포, 대식세포의 변화를 유도한다. 비타민 D의 이러한 기능은 류마티스 관절염, 루푸스, 염증성 장 질환 등과 연관성이 있으며, 피부과 영역에서는 염증성 질환인 아토피 피부염, 건선, 백반증, 원형 탈모등과의 연관성이 알려져 있다. |
| --- |

**2. 연구목적**

| 비타민 D는 반성 염증성 피부질환에서 영향을 미치는 것으로 알려져 있다. 여드름 역시 피부의 만성 염증성 질환이나 여드름에서 비타민 D와읜 연관성은 잘 알려져 있지 않다.  이번 연구에서는 여드름 환자에서 비타민 D 상태를 확인하고 비타민 D를 보충 하였을 때 효과에서 대하여 확인하고자 한다. |
| --- |

**3. 연구기관/ 연구 책임자, 담당자, 공동연구자 성명 및 직명**

연구기관 :충남대학교병원

연구책임자 : 임명 (임상부교수)

소속: 충남대학교병원 피부과

연구담당자 : 임슬기 (전공의)

소속: 충남대학교병원 피부과

**4. 임상연구 대상질환**

임상적으로 진단된 여드름

**5. 시험 대상자 선정/제외기준**

| 목표한 시험대상자 수  1) 총 목표 시험대상자 수 : 여드름 환자 80명, 건강한 대조군 80명  시험대상자 선정기준  1) 20세 이상 35세 이하의 성인 중 임상적으로 진단된 여드름 환자  2) 20세 이상 35세 이하의 성인 중 다른 질환이 없는 건강한 사람  성별  무관함.  시험대상자 제외기준  1) 여드름 치료, 전신 스테로이드제 사용, 비타민 D 보충을 시행 받고 있는 사람  2) 다른 염증성 질환이 동반된 경우 |
| --- |

**6. 임상연구기간**

연구 개시일 : 2014년 11월 1일

대상자 모집 시작일 : 2014년 11월 1일

대상자 모집 종료일 : 2015년 2월 28일

연구 종료일 : 2015년 4월 30일

결과 분석 종료일 : 2015년 5월 31일

**7. 연구방법**

| - 관찰 연구 및 무작위 대조군 연구  1차 연구 : 관찰연구  2차 연구 : 무작위 대조군 연구  - 연구 계획  정보 수집 : 대상자와 관련된 기본 적인 인적 사항을 처음 참여시에 수집한다. (나이, 성별, 체질량지수, 흡연력, 자외선차단제 사용 여부)  1차 연구 : 80명의 여드름 환자와 80명의 건강한 대조군을 대상으로 혈액 검사를 시행하여서 25-hydroxy vitamin D (25(OH)D)의 혈중 농도를 확인한다. 채혈을 정맥에서 이루어지며 채혈된 혈액은 24시간 내에 Roche Cobas e411 (Roche Diagnostics System, Switzerland) 기계를 통해 분석 한다.  2차 연구 : 여드름 환자중에서 비타민 D 결핍상태로 나온 환자를 대상으로 두 그룹으로 무작위 배정을 한다. 첫번째 그룹은 cholecalciferol 1000 IU/day 를 2개월간 복용하며, 두번째 그룹은 동일한 제형의 플라시보 약물을 복용한다. 다른 여드름 치료를 위한 외용제나 약물 사용은 허락되지 않는다. 단 세안제와 보습제는 함께 사용 할 수 있다. |
| --- |

**8. 관찰 항목 및 평가 기준**

| 1차 결과 분석  여드름 환자와 건강한 대조군은 혈액검사를 통해서 25-hydroxy vitamin D (25(OH)D)의 상태를 측정한다. 25(OH)D의 농도에 따라서 3단계로 분류되는데 농도가 20ng/mL 초과인 경우 충분하다고 평가되며, 12-20ng/mL의 경우 충분하지는 않으나 부족하지도 않은 상태로 평가되며, 12ng/mL 미만인 경우는 부족상태로 평가된다. 이러한 기준은 의학연구소의 식품영양 위원회의 권고사항을 바탕으로 작성하였습니다.  2차 경과 분석   1. 여드름의 중증도는 처음 내원당시, 그리고 2,4,8주 차의 사진촬영 결과와 global acne grading system (GAGS) 점수를 이용하여 분석한다. GAGS눈 병변부위를 6군데로 나누어서 이마, 양쪽 볼, 코, 턱, 앞가슴, 등에 대하여 평가하며, 각 부위에 대하여 면적 대비 모발피지선 단위의 분포와 밀도를 고려하여 점수화 한다. 이렇게 나온 점수와 환자의 여드름의 중증도 점수 (면포 1점, 구진 2점, 농포 3점, 결절 4점)를 곱하여 최종 점수를 산출하게 된다. 이렇게 산출된 점수의 합이 1-18점 일 경우 경증, 19-30점일 경우 중등도, 31-38점일 경우 중증, 39 이상일 경우 매우 중증으로 분류한다. 2. 비 염증성 병변(면포)의 개수와 염증성 병변(구진, 농포, 결절)의 개수는 내원시마다 측정되며, 3명의 연구 내용을 알지 못하는 피부과 의사가 개수를 측정한다. |
| --- |

**9. 자료분석과 통계적 방법**

| - 통계 분석은 SPSS version 15 (SSPS Inc., Chicago, IL)프로그램을 사용하여 시행한다. repeated ANOVA를 실시한다. post hoc 분석을 위하여 Mann–Whitney U-test를 시행할 예정이며, Chi-square test, Fisher’s exact test를 이용하여 카테고리별 비교 분석을 시행하겠다. Spearman’s correlation 분석을 이용하여 일치율을 확인하고, 의미 있는 유의수준은 P values < 0.05 설정하여 진행할 예정이다. |
| --- |

**10. 연구결과의 출간 및 보고**

**-** 통계학적 의미를 분석한 후 이에 대한 자료를 바탕으로 논문으로 작성하여 피부과 관련 저널에 보고한다.

**11. 참고문헌**

- Vitamin D status in patients with rosacea. O¨zlem Ekiz et al. Cutan Ocul Toxicol, 2014; 33(1): 60–62

- Serum 25-hydroxyvitamin D deficiency in chinese patients with vitiligo: a case-control study. Xu X et al. PLoS One 2012;7:e52778
- Vitamin D: emerging roles in infection and immunity. Bartly J. et al. Expert Rev Anti Infect Ther 2010;8:1359–1369
- A pilot study assessing the role of 25 hydroxy vitamin D levels in patients

with vitiligo vulgaris. Silverberg JI et al. J Am Acad Dermatol 2010;62:937–941
